# Predictors of in-hospital mortality in patients with multiple wasp stings in southwest China

**DOI:** 10.3389/ftox.2026.1856026

**Published:** 2026-06-12

**Authors:** Yujie Tang, Qin Li, Zhifei Zhang, Yanxia Yi, Zhipeng Zhan

**Affiliations:** 1 Department of Nephrology, Suining Central Hospital, Suining, China; 2 Department of Clinical Laboratory, Suining Central Hospital, Suining, China; 3 Department of Medical Records, Suining Central Hospital, Suining, China

**Keywords:** in-hospital mortality, multiple wasp stings, nomogram, organ injury, predictors, wasp envenomation

## Abstract

**Background:**

Multiple wasp stings can cause severe systemic toxicity, multiple organ dysfunction, and death, but early identification of high-risk patients remains challenging. This study aimed to describe the epidemiological and clinical characteristics of hospitalized patients with multiple wasp stings and to identify independent predictors of in-hospital mortality for early risk stratification.

**Methods:**

In this single-center retrospective study, we analyzed 1,501 patients admitted for multiple wasp stings at Suining Central Hospital between 2011 and 2019. For descriptive analyses, all 1,501 patients were included. For comparative analyses and nomogram development, a subset of 714 adult patients with complete clinical and outcome data was included. Univariate and multivariable logistic regression analyses were performed to identify independent predictors of in-hospital mortality. A nomogram was subsequently constructed, and its discrimination was assessed using the area under the receiver operating characteristic curve (AUC).

**Results:**

In the overall cohort of 1,501 patients, the median age was 52.1 years, 60.2% were male, and the median time from envenomation to hospital admission was 3 days. Major complications included liver injury (16.3%), rhabdomyolysis (15.7%), acute kidney injury (10.3%), myocardial injury (10.2%), hemolysis (4.5%), and pneumonia/acute respiratory distress syndrome (3.1%). Overall, 27.5% of patients required rescue therapy and 7.0% underwent dialysis. Among the 714 adult patients in the analytical cohort, 68 (9.5%) died during hospitalization. Multivariable logistic regression identified serum potassium (OR 2.55, 95% CI 1.59–4.09; p < 0.001), indirect bilirubin (per 10-unit increase; OR 1.15, 95% CI 1.04–1.28; p = 0.005), and prothrombin time (OR 1.14, 95% CI 1.03–1.27; p = 0.015) as independent predictors of in-hospital mortality. The nomogram showed acceptable discrimination, with an AUC of 0.72 (95% CI, 0.65–0.79).

**Conclusion:**

In patients with multiple wasp stings, in-hospital mortality is closely associated with venom-induced multi-organ dysfunction. A nomogram incorporating serum potassium, indirect bilirubin, and prothrombin time may provide a practical and accessible tool for early risk stratification and help clinicians identify high-risk patients for timely intervention.

## 1 Introduction

Wasps, belonging to the order Hymenoptera, are widely distributed worldwide, with an estimated 6,000 species, approximately 200 of which have been reported in China (Yang et al., 2018). As important natural biocontrol agents, they play a pivotal role in maintaining the stability of agricultural ecosystems (Biló et al., 2005). Despite their ecological significance, wasp stings represent a prevalent and underrecognized public health threat. In recent years, the global incidence of wasp stings has increased, accompanied by a substantial rise in associated mortality rates (Pan et al., 2025). Epidemiological studies have indicated that 56%–94% of the global population experiences at least one wasp sting during their lifetime, with approximately 10% of these envenomation events carrying potentially life-threatening risks (Nittner-Marszalska and Cichocka-Jarosz, 2015). Compelling evidence from the World Health Organization (WHO) Mortality Database (Cause of Death Code: X23) shows that 1,691 deaths attributed to stings by wasps and other Hymenoptera species were documented in Europe alone over a 23-year period (1994–2016) (ICD-10, 2019). Regionally, a large-scale outbreak in Shaanxi Province, China, between July and October 2013 resulted in 1,675 wasp sting cases and 42 fatalities (Liu et al., 2016). Additionally, a case series of adult wasp sting patients reported by Zhang et al. revealed a mortality rate of 9.3%, with 10.7% of survivors progressing to chronic kidney disease (CKD) (Zhang et al., 2013). Collectively, these findings underscore the global underestimation of wasp stings as a public health threat, highlighting an urgent need for targeted interventions and greater awareness to mitigate the associated health burden.

Wasp venom comprises a complex mixture of peptides, enzymes, proteins, and other bioactive substances that can induce cellular injury, ranging from localized allergic reactions to multiorgan dysfunction (Luo et al., 2022). Clinically, the manifestations of wasp stings are diverse and can be classified into three types according to the underlying pathogenic mechanisms: direct toxic effects, allergic reactions, and secondary organ damage (Warrell, 2019). Previous studies have reported that the primary therapeutic focus following wasp stings has centered on anti-allergic treatments such as histamine H1/H2 receptor antagonists, corticosteroids, and desensitization protocols (Hamilton, 2010). However, this therapeutic paradigm may be insufficient in the context of mass envenomation, in which systemic toxicity rather than isolated allergic reactions predominates.

An increasing number of recent studies have demonstrated that in mass wasp sting incidents, systemic toxic reactions characterized by multiple organ dysfunction syndrome (MODS) are the predominant manifestations, rather than isolated allergic reactions (Nguyen et al., 2023; Zhang et al., 2025). Southwest China, owing to its geographical location, natural climatic characteristics, and the continuous ecological transition of farmland back to forest, provides a favorable habitat for wasps (Xie et al., 2013). In these regions, the dense distribution of wasps is associated with a significantly higher incidence of MODS than in other areas of China (Wang et al., 2023). However, large-scale studies systematically characterizing hospitalized patients with multiple wasp stings in southwest china remain limited, and practical tools for early prediction of in-hospital mortality are still lacking.

In this single-center retrospective study, we analyzed the clinical data of hospitalized patients with multiple wasp stings admitted to a tertiary hospital in southwest china between 2011 and 2019. We aimed to describe the epidemiological patterns and clinical characteristics of wasp sting-related injuries in this region and to develop a nomogram for predicting in-hospital mortality based on readily available laboratory markers. These findings may contribute to early risk stratification and clinical decision-making in high-risk patients.

## 2 Material and methods

### 2.1 Clinical data collection

This was a single-center retrospective study conducted at Suining Central Hospital, a tertiary hospital in southwest china. A total of 1,501 patients with multiple wasp stings were admitted to Suining Central Hospital between January 2011 and December 2019. Multiple wasp stings were defined as the presence of two or more visible sting wounds caused by wasps. The diagnosis was established based on a definite history of wasp exposure, visible sting wounds, compatible local and/or systemic clinical manifestations, and relevant laboratory abnormalities when present. Local manifestations included pain, erythema, swelling, and pruritus around the sting sites. Systemic manifestations included dizziness, nausea, vomiting, fever, hematuria, oliguria or anuria, jaundice, hypotension, altered consciousness, or other signs of organ dysfunction. Patients with only local reactions or mild systemic symptoms but without major organ dysfunction were classified as mild cases, whereas those with organ dysfunction or systemic complications were classified as severe cases. No formal severity scoring system was applied in this retrospective study.

**FIGURE 1:**
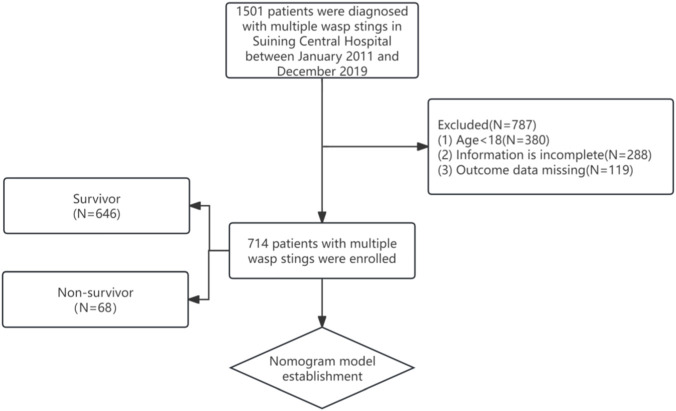
Research Flowchart.

During the study period, 714 adult patients with available key clinical and outcome data were included in the detailed analysis. A total of 787 patients were excluded, including 380 patients aged <18 years, 288 patients with incomplete clinical information, and 119 patients with missing outcome data. The screening and enrollment process is illustrated in Figure 1. For the descriptive analysis of the overall clinical profile and complication burden, data from all 1,501 patients were used whenever available. For the comparative analysis between survivors and non-survivors and for the development of the in-hospital mortality prediction nomogram, only the 714 adult patients with available key clinical and outcome data were included. Eligible patients were identified through screening of the hospital’s electronic medical record (EMR) system and manual review of medical record front pages. Data were extracted using a standardized data collection form, and all required clinical and demographic information was retrieved from medical records archived in the Department of Medical Records. All collected data were subsequently entered into a dedicated database for statistical analysis.

As this was a single-center retrospective study, no formal prospective sample size calculation was performed before patient enrollment. Instead, all eligible patients with multiple wasp stings admitted during the predefined study period were consecutively screened. The final sample size was determined by the number of adult patients with available key clinical information and outcome data.”

### 2.2 Ethics approval and consent to participate

This retrospective study was approved by the Institutional Review Board (IRB) of Suining Central Hospital (Approval No. KYLLKS20260049). All procedures involving human participants were conducted in accordance with the ethical standards of the institutional and/or national research committees, as well as the 1964 Declaration of Helsinki and its subsequent amendments or other comparable ethical standards. Given the retrospective design of the study and the use of medical records from patients who had already completed treatment, the IRB waived the requirement for informed consent.

### 2.3 Clinical characteristics

The following clinical information was collected for each patient: presence of hematuria, hypotension, or oliguria/anuria on admission; number of stings; time interval between sting and hospitalization; duration of oliguria/anuria; other clinical manifestations (including vomiting, hematemesis, arrhythmia, heart failure, pulmonary edema, altered consciousness, and other complications); duration of hospital stay; in-hospital mortality; and cause of death.

### 2.4 Definitions of clinical outcomes and key terms

Key clinical terms and complications during hospitalization were identified according to the following operational definitions:

#### 2.4.1 Multiple wasp stings

The presence of two or more visible sting wounds caused by wasps.

#### 2.4.2 Rescue therapy

The implementation of at least one of the following life-sustaining interventions: mechanical ventilation, blood purification (continuous renal replacement therapy or hemoperfusion), cardiopulmonary resuscitation, or the administration of vasoactive agents (e.g., norepinephrine) to maintain hemodynamic stability.

#### 2.4.3 Severe cases

Patients presenting with acute organ dysfunction (e.g., acute kidney injury, liver injury, acute respiratory distress syndrome), severe metabolic complications (e.g., rhabdomyolysis, coagulopathy), or life-threatening systemic reactions (e.g., anaphylactic shock) resulting from wasp stings.

#### 2.4.4 Rhabdomyolysis

Serum creatine kinase (CK) > 5 times the upper limit of normal (ULN), with or without compatible clinical manifestations such as muscle pain, muscle weakness, or dark urine. Elevated serum or urine myoglobin, when measured, provided additional supportive evidence.

#### 2.4.5 Liver injury

Peak alanine aminotransferase (ALT) or aspartate aminotransferase (AST) level >3 times the ULN during hospitalization.

#### 2.4.6 Myocardial injury

High-sensitivity cardiac troponin I (hs-cTnI) exceeding the 99th percentile upper reference limit (URL).

#### 2.4.7 Coagulopathy

Prothrombin time (PT) > 17.5 s or activated partial thromboplastin time (APTT) > 55.0 s (reference ranges in our laboratory: PT 11.0–14.0 s, APTT 25.0–45.0 s).

#### 2.4.8 Hemolysis

Evidence of red blood cell destruction, based on the presence of at least two of the following standard laboratory abnormalities: elevated plasma free hemoglobin (>10 mg/dL or >1.55 μmol/L), hemoglobinuria, decreased or absent haptoglobin (<0.3 g/L), elevated lactate dehydrogenase (LDH > 2times the ULN), or elevated indirect bilirubin (>1.2 mg/dL or >20.5 μmol/L).

#### 2.4.9 Anaphylactic shock

Acute onset of hypotension (systolic blood pressure <90 mmHg or a decrease >30% from baseline) with involvement of at least one additional organ system (e.g., respiratory: wheezing, stridor, hypoxemia; cutaneous: urticaria, angioedema, flushing; gastrointestinal: abdominal pain, vomiting).

#### 2.4.10 Non-anaphylactic shock

Hypotension (systolic blood pressure <90 mmHg or a decrease >30% from baseline) attributed to venom-induced vasodilation or capillary leak, without accompanying IgE-mediated allergic manifestations.

#### 2.4.11 Acute kidney injury (AKI)

Diagnosed according to the KDIGO 2012 serum creatinine criteria, defined as an increase in serum creatinine by ≥ 0.3 mg/dL (≥26.5 μmol/L) within 48 h or an increase to ≥1.5 times baseline within the prior 7 days. Baseline serum creatinine was defined as the most recent pre-admission value when available; otherwise, the earliest post-admission value was used. The urine output criterion was not applied due to incomplete hourly urine output documentation in this retrospective study.

These operational definitions were prespecified before data analysis and applied consistently across all patients.

### 2.5 Treatment

All patients received glucocorticoid and antihistamine therapy. Additional epinephrine was administered to those with severe allergic reactions. Blood purification therapy was indicated for patients with any of the following conditions: (1) MODS; (2) anuria or oliguria lasting for more than 2 days; (3) acute pulmonary edema; (4) severe hyperkalemia (serum potassium >6.5 mmol/L); (5) severe metabolic acidosis (pH < 7.2); or (6) dark brown urine accompanied by a daily increase in blood urea nitrogen (BUN) ≥14.3 mmol/L and elevated Scr.

### 2.6 Statistical analysis

Normally distributed continuous data were expressed as mean ± standard deviation (SD), and comparisons between the two groups were performed using an unpaired t-test. Non-normally distributed continuous data were presented as median and interquartile range (IQR), and the Mann–Whitney U-test was applied. Categorical variables were presented as frequencies or proportions, and differences between groups were compared using the chi-square test or Fisher’s exact test, as appropriate.

Prior to analysis, data integrity was assessed by evaluating the proportion of missing values for each candidate variable. Variables with more than 20% missing values were excluded to ensure model reliability. For the remaining variables, multiple imputation was performed to address missing data and minimize potential bias.

Univariate logistic regression was conducted to screen for potential predictors of in-hospital mortality. Variables with *p* < 0.1 or established clinical significance were entered into the multivariable model. Multicollinearity was assessed using the variance inflation factor (VIF), with VIF >5 used to identify and exclude highly redundant variables. Independent predictors were identified using a multivariable logistic regression model with the forward selection method, and a nomogram was subsequently constructed for risk visualization. Internal validation was performed using the bootstrap method with 1,000 resamples to evaluate model stability and quantify optimism. Calibration curves were plotted to assess agreement between predicted probabilities and actual outcomes. The discriminative ability of the nomogram was evaluated using receiver operating characteristic (ROC) curve analysis, with the area under the curve (AUC) used to quantify predictive accuracy. A two-tailed *p* < 0.05 was considered statistically significant. All statistical analyses were performed using Stata version 15.0.

## 3 Results

### 3.1 Demographic and clinical characteristics

A total of 1,501 patients were admitted for multiple wasp stings during the study period. The mean age was 44.4 ± 25.7 years, and 904 patients (60.2%) were male. The median time from envenomation to hospital admission was 3 days (IQR, 1–5 days). Across the entire cohort, the major complications recorded were as follows: liver injury in 244 cases (16.3%), rhabdomyolysis in 235 cases (15.7%), acute kidney injury (AKI) in 155 cases (10.3%), myocardial injury in 153 cases (10.2%), hemolysis in 68 cases (4.5%), pneumonia/acute respiratory distress syndrome (ARDS) in 46 cases (3.1%), gastrointestinal (GI) bleeding in 15 cases (1.0%), and encephalopathy in 7 cases (0.5%). A total of 413 patients (27.5%) required rescue therapy, and 105 patients (7.0%) underwent dialysis (Table 1).

**TABLE 1:** Baseline characteristics of the study population (n = 1,501).

Characteristic	Value
Demographic characteristics	
Male sex, n (%)	904 (60.2)
Age (years), median (IQR)	52.1 (17.2–65.4)
<18, n (%)	380 (25.3)
≥18s, n (%)	1121 (74.7)
Duration of hospitalization, days, median (IQR)	3 (2–6)
Clinical complications	
Acute kidney injury, n (%)	155 (10.3)
Rhabdomyolysis, n (%)	235 (15.7)
Hemolysis, n (%)	68 (4.5)
Pneumonia/ARDS, n (%)	46 (3.1)
Gastrointestinal bleeding, n (%)	15 (1.0)
Liver injury, n (%)	244 (16.3)
Myocardial injury, n (%)	153 (10.2)
Encephalopathy, n (%)	7 (0.5)
Dialysis, n (%)	105 (7.0)
Rescue, n (%)	413 (27.5)
Outcome	
In-hospital death among adults, n (%)	68 (9.5)*

(Abbreviations: ARDS, acute respiratory distress syndrome; IQR, interquartile range.

Data are presented as n (%) or median (IQR). *, The percentage of in-hospital death was calculated among adult patients with available outcome data(n = 714).

The initial laboratory findings of the study population are summarized in Table 2. White blood cell (WBC) count, creatine kinase (CK), and glucose levels were elevated, whereas aspartate aminotransferase (AST) and indirect bilirubin (IBIL) were slightly above the normal reference ranges.

**TABLE 2:** Laboratory results of the study patients (n = 714).

Characteristic	N	Values
WBC count (3.5–9.5 ×10^9^/L)	631	14.0 ± 6.2
Hemoglobin (130–175 g/L)	631	134.0 (120.0–145.0)
Urea (2.86–8.20 mmol/L)	642	6.2 (5.0–7.8)
Creatinine (59–104 μmol/L)	642	68.0 (56.0–82.0)
APTT (25–45 s)	594	48.8 (34.8–89.0)
PT (11–14 s)	594	13.9 ± 1.6
AST (15–40 U/L)	648	37.0 (26.0–68.0)
ALT (9–50 U/L)	648	24.0 (17.0–42.0)
TBIL (5.1–28 μmol/L)	648	16.1 (9.6–31.0)
IBIL (0–18 μmol/L)	648	9.8 (6.0–21.2)
Albumin (40–55 g/L)	648	41.2 ± 4.5
CK (38–174 U/L)	647	227.0 (115.5–553.5)
LDH (124–225 U/L)	647	223.0 (186.5–325.5)
Glucose (3.89–6.11 mmol/L)	564	6.8 (5.5–8.6)
Potassium (3.5–5.3 mmol/L)	638	3.6 ± 0.5
Sodium (137–147 mmol/L)	638	141.8 ± 3.9

APTT, activated partial thromboplastin time; PT, prothrombin time; BUN, blood urea nitrogen; AST, aspartate aminotransferase; ALT, alanine aminotransferase; TBIL, total bilirubin; IBIL, indirect bilirubin; CK, creatine kinase; LDH, lactate dehydrogenase.

### 3.2 Complications and laboratory findings between survivors and non-survivors

As shown in Table 3, the proportions of patients with rhabdomyolysis (p = 0.003), hemolysis (p = 0.037), pneumonia/ARDS (p = 0.001), and encephalopathy (p = 0.003) were significantly higher in the non-survivor group than in the survivor group. Furthermore, peripheral blood parameters, organ function, electrolyte balance, and coagulation profiles were compared (Table 4). The non-survivor group exhibited significantly higher levels of WBC count (p = 0.001), blood urea nitrogen (BUN) (p = 0.009), creatinine (p = 0.001), total bilirubin (TBIL) (p < 0.001), IBIL (p < 0.001), AST (p < 0.001), alanine aminotransferase (ALT) (p = 0.002), lactate dehydrogenase (LDH) (p < 0.001), glucose (p = 0.007), and potassium (p < 0.001). These findings indicate that in-hospital mortality was associated with more severe organ injury, electrolyte imbalance, and coagulation dysfunction.

**TABLE 3:** Comparison of complications between survival and non-survivors groups (n = 714).

Complications	Survived (n = 646)	Died (n = 68)	*p*-Value
Acute kidney injury, n (%)	88 (13.6)	15 (22.1)	0.06^a^
Rhabdomyolysis, n (%)	127 (19.7)	24 (35.3)	0.003^a^
Hemolysis, n (%)	39 (6.0)	9 (13.2)	0.037^b^
Pneumonia/ARDS, n (%)	21 (3.3)	9 (13.2)	0.001^b^
GI bleeding, n (%)	10 (1.5)	0 (0.0)	0.61^b^
Liver injury, n (%)	136 (21.1)	17 (25.0)	0.451^a^
Myocardial injury, n (%)	82 (12.7)	9 (13.2)	0.899^a^
Encephalopathy, n (%)	1 (0.2)	3 (4.4)	0.003^b^

ARDS: acute respiratory distress syndrome; GI, bleeding: gastrointestinal bleeding.

a Pearson’s chi-squared test (two-sided).

^b^ Fisher’s exact test.

**TABLE 4:** Comparison of laboratory results between survival and non-survivors group (n = 714).

Laboratory parameters	Survived (n = 646)			Died (n = 68)			P-value*
	N	Median	IQR	N	Median	IQR	
WBC count (×10^9^/L)	574	12.8	9.4–16.9	57	15.9	11.2–22.5	0.001
Hemoglobin (g/L)	574	134.0	121.0–145.0	57	131.0	119.0–145.0	0.295
BUN (mmol/L)	585	6.1	5.0–7.8	57	7.0	5.6–9.0	0.009
Creatinine (µmol/L)	585	67.0	55.0–80.0	57	75.0	63.0–93.0	0.001
APTT (sec)	537	47.7	34.7–86.5	57	65.8	37.7–113.8	0.027
PT (sec)	537	13.7	13.0–14.5	57	13.9	13.1–15.3	0.085
AST (U/L)	589	36.0	26.0–64.0	59	60.0	28.5–482.0	<0.001
ALT (U/L)	589	24.0	17.0–38.0	59	34.0	19.5–199.5	0.002
TBIL (µmol/L)	589	15.5	9.5–28.8	59	25.4	12.6–66.3	<0.001
IBIL (µmol/L)	589	9.6	5.8–19.2	59	20.1	8.4–52.3	<0.001
Albumin (g/L)	589	41.3	38.5–44.0	59	42.3	39.9–45.2	0.073
CK (U/L)	589	224.0	115.0–516.0	58	273.0	119.0–804.8	0.252
LDH (U/L)	589	220.0	185.0–308.0	58	271.0	200.5–1474.0	<0.001
Glucose (mmol/L)	510	6.7	5.5–8.5	54	8.0	6.3–9.9	0.007
Potassium (mmol/L)	581	3.5	3.2–3.8	57	3.8	3.5–4.2	<0.001
Sodium (mmol/L)	581	142.4	140.0–144.2	57	142.0	140.8–144.1	0.973

APTT, activated partial thromboplastin time; PT, prothrombin time; BUN, blood urea nitrogen; AST, aspartate aminotransferase; ALT, alanine aminotransferase; TBIL, total bilirubin; IBIL, indirect bilirubin; CK, creatine kinase; LDH, lactate dehydrogenase. Mann–Whitney U-test, two-tailed. * indicates P-values calculated by Mann-Whitney U-test, two-tailed.

### 3.3 Independent risk factors for in-hospital mortality and nomogram construction

**FIGURE 2:**
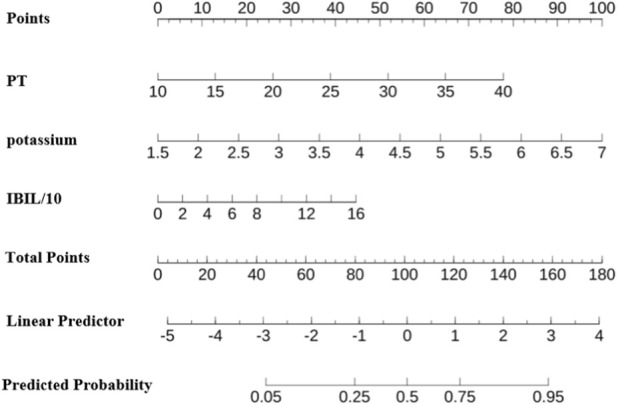
Nomogram for Predicting In - hospital Mortality from Wasp Stings.

**FIGURE 3:**
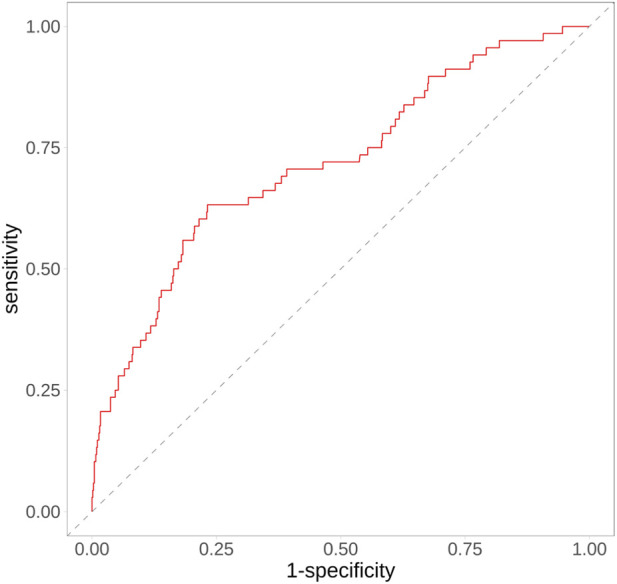
ROC curves of nomograms for predicting In - hospital Mortality from Wasp Stings.

Multivariable logistic regression analysis identified serum potassium (OR = 2.55, 95% CI: 1.59–4.09, p < 0.001), IBIL (per 10-unit increase: OR = 1.15, 95% CI: 1.04–1.28, p = 0.005), and PT (OR = 1.14, 95% CI: 1.03–1.27, p = 0.015) as independent predictors of in-hospital mortality (Table 5). Based on these independent predictors, a nomogram was developed to visualize and quantify the risk of in-hospital mortality in patients with wasp stings (Figure 2). The model demonstrated good discriminative ability, with an area under the receiver operating characteristic (ROC) curve (AUC) of 0.72 (95% CI: 0.65–0.79), effectively distinguishing between non-survivors and survivors (Figure 3).

**TABLE 5:** Univariable and multivariable binary logistic regression analyses of predictors of in-hospital mortality in patients with multiple wasp stings.

Variables	Univariable analysis			Multivariable analysis		
	OR	95% CI	P Value	OR	95% CI	P value
Potassium (mmol/L)	1.67	1.26–2.22	<0.001	2.55	1.59,4.09	<0.001
PT (sec)	1.17	1.05–1.31	0.005	1.14	1.03,1.27	0.015
IBIL/10	1.10	1.00–1.22	0.001	1.15	1.04,1.28	0.005
WBC count (×10^9^/L)	1.03	1.01–1.06	0.011	-	-	-
Hemoglobin (g/L)	1.00	0.99–1.00	0.268	-	-	-
BUN (mmol/L)	1.02	0.99–1.06	0.190	-	-	-
Creatinine (µmol/L)	1.00	1.00–1.00	0.283	-	-	-
APTT (sec)	1.00	1.00–1.01	0.070	-	-	-
AST (U/L)	1.00	1.00–1.00	0.011	-	-	-
ALT (U/L)	1.00	1.00–1.00	0.003	-	-	-
TBIL (µmol/L)	1.01	1.00–1.02	<0.001	-	-	-
Albumin (g/L)	1.02	0.98–1.05	0.332	-	-	-
CK (U/L)	1.00	1.00–1.00	0.848	-	-	-
LDH (U/L)	1.00	1.00–1.00	0.004	-	-	-
Glucose (mmol/L)	1.02	0.98–1.06	0.324	-	-	-
Sodium (mmol/L)	1.00	0.96–1.04	0.989	-	-	-

OR, odds ratio; CI, confidence interval; WBC, white blood cell; BUN, blood urea nitrogen; APTT, activated partial thromboplastin time; PT, prothrombin time; AST, aspartate aminotransferase; ALT, alanine aminotransferase; TBIL, total bilirubin; IBIL, indirect bilirubin; CK, creatine kinase; LDH, lactate dehydrogenase. IBIL/10 indicates indirect bilirubin divided by 10. Dashes indicate that the variables were not retained in the final multivariable logistic regression model.

## 4 Discussion

Wasp stings can manifest as a broad spectrum of clinical conditions, ranging from localized skin reactions to life-threatening systemic toxicity and multi-organ dysfunction (Ruwanpathirana and Priyankara, 2022). Although reported worldwide, such incidents are particularly prevalent in developing regions, where swarm attacks occur more frequently (Loh and Tan, 2012). Southwest China, characterized by its unique ecological environment, provides a favorable habitat for wasps, resulting in frequent sting incidents and posing a significant public health challenge (P. Chinese Society Of Toxicology et al., 2018). In this retrospective study, we analyzed clinical data from 1,501 patients admitted for multiple wasp stings over a 9-year period (2011–2019). Among them, 714 adult patients with complete clinical and outcome data were included in the detailed comparative analysis and nomogram development. To our knowledge, this represents one of the largest single-center cohorts of patients with multiple wasp stings reported from southwest china to date. Moreover, this is the first study from this region to develop a nomogram for the early prediction of in-hospital mortality. Using multivariable logistic regression analysis, we identified serum potassium, IBIL, and PT as independent predictors of in-hospital mortality.

Wasp venom is composed of a complex mixture of bioactive components, including melittin, mast cell degranulating peptide (MCDP), hyaluronidase, and phospholipase A2 (PLA2). These components are key mediators of cellular injury and tissue damage (Chen et al., 2016; Klotz et al., 2009). Toxic reactions induced by these venom components are dose-dependent and typically occur after multiple simultaneous stings (Giovannini et al., 2024). Common mild manifestations include erythema, nausea, vomiting, diarrhea, headache, dizziness, and fever (Guzel et al., 2016). However, severe complications often develop within hours to days after envenomation, the severity of which is closely associated with the number of stings (Vasconez-Gonzalez et al., 2025; Fitzgerald and Flood, 2006). Consistent with these pathophysiological features, our study revealed severe clinical courses in a significant proportion of patients, with 68 deaths (9.5%) occurring among the fully evaluated cohort. Across the study population, the incidences of major complications—including liver injury (16.3%), rhabdomyolysis (15.7%), acute kidney injury (10.3%), myocardial injury (10.2%), and hemolysis (4.5%) were notably high. Additionally, 413 patients required rescue interventions. These findings suggest that multiple organ damage caused by venom-induced toxicity is a major contributor to disease severity and mortality in massive envenomation, whereas simple immunoglobulin E (IgE)-mediated allergic anaphylaxis may not fully explain the clinical deterioration observed in hospitalized patients with severe wasp stings.

Previous studies have also compared clinical characteristics between survivors and non-survivors after wasp stings. In general, non-survivors have been reported to present with more severe systemic toxic reactions and a higher incidence of organ dysfunction, including acute kidney injury, hepatic dysfunction, rhabdomyolysis, hemolysis, coagulation abnormalities, myocardial injury, shock, and MODS (Xie et al., 2013; Wu et al., 2024; Zhang et al., 2024). These findings are broadly consistent with our results, in which non-survivors showed more severe laboratory abnormalities and organ injury than survivors. In particular, prior research has frequently identified established markers of organ dysfunction, such as elevated serum creatinine, aminotransferases, creatine kinase, lactate dehydrogenase, and evidence of MODS, as important indicators associated with poor outcomes after wasp stings. Our study supports the view that progressive systemic venom toxicity and subsequent multi-organ dysfunction play central roles in fatal wasp envenomation.

Nevertheless, our findings differed in several respects from previous reports. Earlier studies have identified the number of stings, acute kidney injury, liver dysfunction, rhabdomyolysis, and MODS as major predictors of mortality after wasp stings (Wang et al., 2023; Lv and Ge, 2025). In contrast, our multivariable analysis identified serum potassium, IBIL, and PT as independent predictors of in-hospital mortality. These parameters may represent early and readily available laboratory indicators reflecting key pathophysiological processes after wasp envenomation, including tissue injury and rhabdomyolysis, intravascular hemolysis, and coagulation disturbance. This discrepancy may be partly explained by differences in study populations, regional wasp species, inclusion criteria, timing of hospital admission, treatment strategies, and definitions of organ dysfunction. Moreover, the number of stings does not necessarily reflect the actual amount of venom injected, venom composition, individual susceptibility, or the delay between envenomation and treatment. Therefore, our findings complement previous studies by suggesting that simple biochemical and coagulation parameters may provide additional prognostic information beyond traditional markers of established organ failure.

Hyperkalemia is a well-recognized consequence of massive tissue damage and renal impairment (Kim et al., 2023; Hunter and Bailey, 2019). In our study, serum potassium levels were significantly elevated in the non-survivor group. Wasp venom directly disrupts myocyte membranes, triggering rhabdomyolysis and the subsequent massive release of intracellular potassium into the systemic circulation (Mendoza-Razo et al., 2025). Our multivariate analysis confirmed that elevated potassium is an independent predictor of poor prognosis, aligning with findings from similar studies in other regions (Mendoza-Razo et al., 2025). This suggests that serum potassium levels can serve as a robust proxy for the extent of venom-induced muscle and tissue necrosis, requiring immediate clinical attention to prevent fatal cardiac events.

Hemolysis also plays a pivotal role in the progression of wasp-sting-related injury. The significant elevation of IBIL observed in our non-surviving patients likely results from severe intravascular hemolysis, where PLA2 synergizes with venom cations to compromise erythrocyte membrane integrity (Silva et al., 2017). We identified IBIL as an independent risk factor for mortality, reinforcing the consensus that hemolysis is a critical pathological driver in these cases. Furthermore, the massive release of hemoglobin and bilirubin contributes to the development of AKI through pigment nephropathy and tubular obstruction, a correlation well-supported by previous literature (Zheng et al., 2023).

Coagulopathy, particularly reflected by prolonged PT, emerged as another key predictor of mortality in our cohort. Wasp venom toxins can directly damage the vascular endothelium, activate the extrinsic coagulation pathway, and consume coagulation factors, potentially progressing to disseminated intravascular coagulation (DIC) in severe cases (Petroianu et al., 2000). The identification of PT as a risk factor highlights that early disturbances in the coagulation cascade may serve as a “red flag” for the imminent onset of MODS.

By integrating potassium, IBIL, and PT, our nomogram offers a practical and visualized tool for risk stratification with good discriminative ability (AUC = 0.72). Regular monitoring of these markers enables emergency and intensive care clinicians to identify high-risk patients early and implement targeted interventions—such as aggressive fluid resuscitation, timely blood purification modalities (e.g., hemoperfusion or continuous renal replacement therapy), and targeted coagulopathy management—before irreversible multi-organ failure occurs.

Despite the strength of our large sample size, several limitations persist in this study. First, the retrospective, single-center design inherently introduces potential selection bias. Second, the exact number of wasp stings per patient was often unavailable due to the emergency nature of the events, precluding a dose-response analysis of venom toxicity. Third, AKI was diagnosed using the serum creatinine-based criteria of the KDIGO 2012 guidelines because accurate hourly urine output data were not consistently documented in this retrospective cohort. Therefore, the urine output criterion was not incorporated into the primary AKI definition, which may have led to underestimation of AKI incidence, particularly early-stage cases. Fourth, the lack of dynamic, longitudinal monitoring of these laboratory markers limits our ability to track real-time disease progression trajectories. Finally, although internally validated using bootstrap resampling, the proposed nomogram requires external validation in independent, multi-center prospective cohorts to confirm its generalizability and clinical utility.

## 5 Conclusion

In-hospital mortality following multiple wasp stings is closely associated with venom-induced toxic complications, particularly rhabdomyolysis and hemolysis. Serum potassium, indirect bilirubin (IBIL), and prothrombin time (PT) were identified as independent predictors of death. The nomogram based on these variables may provide a practical and accessible tool for the early identification of high-risk patients. Clinicians should prioritize monitoring of electrolyte balance, hemolytic activity, and coagulation function to optimize treatment strategies and improve outcomes in patients with severe wasp envenomation.

## Data Availability

The raw data supporting the conclusions of this article will be made available by the authors, without undue reservation.
